# Information Measure for Long-Range Correlated Sequences: the Case of the 24 Human Chromosomes

**DOI:** 10.1038/srep02721

**Published:** 2013-09-23

**Authors:** A. Carbone

**Affiliations:** 1 Politecnico di Torino, Italy; 2 ISC-CNR, Unità Università ‘La Sapienza’ di Roma, Italy; 3 ETH Zurich, Switzerland

## Abstract

A new approach to estimate the Shannon entropy of a long-range correlated sequence is proposed. The entropy is written as the sum of two terms corresponding respectively to power-law (*ordered*) and exponentially (*disordered*) distributed blocks (clusters). The approach is illustrated on the 24 human chromosome sequences by taking the nucleotide composition as the relevant information to be encoded/decoded. Interestingly, the nucleotide composition of the *ordered* clusters is found, on the average, comparable to the one of the whole analyzed sequence, while that of the *disordered* clusters fluctuates. From the information theory standpoint, this means that the power-law correlated clusters carry the same information of the whole analysed sequence. Furthermore, the fluctuations of the nucleotide composition of the disordered clusters are linked to relevant biological properties, such as segmental duplications and gene density.

Complex systems are probed by observing a relevant quantity over a certain temporal or spatial range, yielding long-range correlated sequences or arrays, with the remarkable feature of displaying ‘ordered’ patterns, which emerge from the seemingly random structure. The degree of ‘order’ is intrinsically linked to the information embedded in the patterns, whose extraction and quantification might add clues to many complex phenomena^1^,^2^,^3^,^4^,^5^,^6^,^7^,^8^,^9^,^10^,^11^,^12^.

In this work, an information measure for long-range correlated sequences, worked out from a partition of the sequence into *clusters* according to the method proposed in^8^,^9^, is put forward. The clusters are characterized by their length *ℓ*, duration *τ* and area 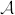, obeying power-law probability distributions, with a cross-over to an exponential decay at large size. The probability distribution function of the lengths is considered to estimate the Shannon (block) entropy *S*(*ℓ*) of the clusters. The entropy can be written as the sum of three terms, respectively constant, logarithmic and linear function of the cluster length. The clusters with dominant logarithmic term of the entropy are power-law correlated and correspond to ‘ordered’ structures, while those with dominant linear term are exponentially distributed and correspond to ‘disordered’ structures. The information measure is illustrated by analyzing the 24 nucleotide sequences of the human chromosomes. Each sequence is first mapped to a fractional Brownian walk (the so-called DNA walk). Then, the probability distribution function *P*(*ℓ*) and the entropy *S*(*ℓ*) of the DNA clusters are estimated by adopting the proposed approach.

It is worth recalling that the investigation of the block entropy of a signal was originally motivated by cryptography. Claude Shannon attempt was aimed at encoding information in ways that still allowed recovery by the receiver, the main question to be answered being: ‘How the signal can be compressed in elementary messages which still contain the relevant information to be communicated?’. The approach proposed in this work represents a possible answer. Furthermore, this question recalls the concept of Kolmogorov complexity *KC*(*ℓ*) which quantifies the interplay of randomness/determinism of the strings output of a computational program. The Kolmogorov complexity is quantified in terms of the minimal length of the program that can still generate a random string. It can be demonstrated that the length of the program, which is defined case-by-case in the specific computational framework, is comparable to the length of the string plus a constant, and varies as the logarithm of the length of the string itself.

From the information theory standpoint, the present work shows that by taking the nucleotide composition of the whole sequence as the relevant information to be transmitted from the source to the receiver, the whole sequence is encoded in blocks (clusters), which are able to transmit the same information of the whole sequence if they are power-law correlated. Specifically, it is shown that the power-law correlated clusters are characterized by a nucleotide content, purine-pyrimidine pairs (GC)% and (AT)%, on the average equal to the value of the whole chromosome sequence under analysis. Conversely, the exponentially correlated clusters are characterized by a percentage of purine-pyrimidine pairs exhibiting fluctuations around the value taken by the whole sequence. Interestingly, the standard deviationof the cluster composition fluctuations for each of the 24 chromosomes is correlated to biologically relevant properties, such as duplication frequency and gene density. It is worthy of remark that the nucleotide composition is taken as a case study for the illustration of the implementation and meaning of the proposed entropy measure, but it is not the only biologically relevant information carried by a DNA sequence.

## Results

The entropy of a sequence, coded in blocks, has been extensively studied since its introduction by Shannon (see e.g.^2^,^3^,^4^,^5^, and Refs. therein). The practical application of the Shannon entropy concept requires a symbolic representation of the data, obtained by a suitable partition transforming the continuous phase-space into disjoint sets. As discussed in^5^, the construction of the optimal partition is not a trivial task, being crucial to effectively discriminate between randomness/determinism of the encoded/decoded data. The method commonly adopted for partitioning a sequence and estimating its entropy is based on a uniform division in blocks having equal length *ℓ*. Then the entropy is estimated over subsequent partition corresponding to different blocks lengths *ℓ*. The novelty of the present work resides in the method used for partitioning the sequence which directly yields power-law or exponential distributed blocks (clusters). This is a major advantage, as it allows one to straightforwardly separate the set of inherently correlated/uncorrelated blocks along the sequence.

**Figure 1:**
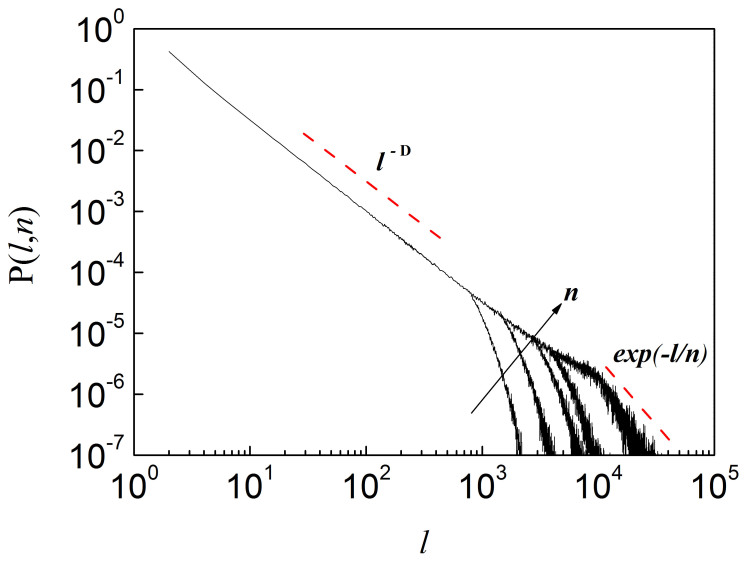
Cluster Length Probability Distribution. Probability distribution function *P*(*ℓ*, *n*) of cluster lengths for a sequence with *H* ≈ 0.6 and *L* = 2 ^20^. The moving average windows are *n* = 500, *n* = 1000, *n* = 2000, *n* = 3000 and *n* = 10000 (from left to right). As *n* increases, *P*(*ℓ*, *n*) becomes broader. The slope of the distribution becomes steeper for *ℓ* > *n*, corresponding to the onset of finite-size effects and exponentially decaying correlation.

A random sequence *y*(*x*) can be partitioned in elementary clusters by the intersection with the moving average 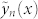 where *n* is the size of the moving window. The clusters correspond to the regions bounded by *y*(*x*) and 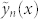 between two subsequent crossings points *x_c_*(*i*) and *x_c_*(*i* + 1)^8^. The intersection between *y*(*x*) and 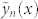 produces a *generating* partition, yielding different sequences of clusters for different values of *n*. The probability distribution function *P*(*ℓ*, *n*) of the lengths *ℓ* for each *n* can be obtained by counting the clusters 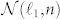,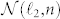,…,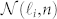 respectively with length *ℓ*_1_, *ℓ*_2_, …, *ℓ_i_*. By doing so, one obtains^8^: 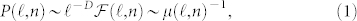
where *D* = 2 − *H* and *H* indicate respectively the fractal dimension and the Hurst exponent of the sequence. The exponent *H* is widely used for quantifying long-range correlations (power-law decaying) as opposed to short-range (exponentially decaying) correlations in many complex systems. The Hurst exponent has been estimated for the 24 chromosome sequences, as reported in the 3*^rd^* of Table 1. The occurrence of long-range correlations means that the nucleotides are organized along the sequences in similar way, a fact that can be defined as *compositional self-similarity* of the chromosomes. The function 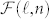 in equation 1 can be taken of the form: 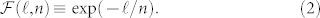

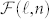
 accounts for the drop-off of *P*(*ℓ*, *n*) due to finiteness of *n* when *ℓ* ≫ *n*. The quantity *μ*(*ℓ*, *n*) ~ *ℓ^D^* exp(*ℓ*/*n*) is proportional to the size of the subsets spanned by the random walkers which ranges from a line proportional to *ℓ* for *H* = 1 to a square proportional to *ℓ*^2^ for *H* = 0 for *n* > *ℓ*. The probability distribution function *P*(*ℓ*, *n*) is shown in Fig. 1 for a wide range of *n* values, estimated for a long range correlated series with Hurst exponent *H* ≈ 0.6. For *n* → 1, the lengths *ℓ* of the elementary clusters are centered around a single value. When *n* increases, a broader range of lengths is obtained and, consequently, *P*(*ℓ*, *n*) spreads over all values.

The Shannon entropy is defined as^2^,^3^,^4^,^5^: 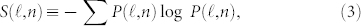
where the sum is performed over the number of elementary clusters with length *ℓ* obtained by the intersection with the moving average for each *n*. This number ranges from 1 to *μ*(*ℓ*, *n*)^–1^ depending on how many clusters are generated by the intersection with the moving average. The value 1 is obtained when only one cluster with length *ℓ* is found in the partition. As already noted, the standard method for partitioning a sequence and estimating its entropy is by splitting the sequence into a set of disjoint blocks with equal length *ℓ*. Conversely, in the present work, the intersections of the sequence with the moving average generate a set of disjoint blocks with a broad distribution of lengths *ℓ* corresponding respectively to power-law or exponential correlation. This particular partition retains the determinism/randomness of the blocks by simply varying *n*, an aspect intimately related to the Kolmogorov complexity concept.

By using equations (1) and (3), the cluster entropy writes: 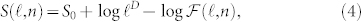
which, after taking into account equation (2), becomes: 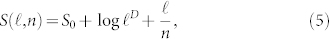
where *S*_0_ is a constant, log *ℓ^D^* is related to the term *ℓ*^–*D*^ and *ℓ*/*n* is related to the term 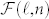.

To clarify the meaning of the terms appearing in equation (5), it is worthy of remarking that for *isolated systems*, the entropy increase *dS* is related to the irreversible processes spontaneously occurring within the system. The entropy tends to a constant value as a stationary state is asymptotically reached (*dS* ≥ 0). For *open systems* interacting with their environment, the increase is given by a term *dS_int_*, due to the irreversible processes spontaneously occurring within the system, and a term *dS_ext_* due to the irreversible processes arising through the external interactions. The term log *ℓ^D^* in equation (5) should be interpreted as the intrinsic entropy *S_int_*. It is indeed independent of *n*, i.e. it is independent of the method used for partitioning the sequence, which plays here the role of the external interaction. The logarithmic term is of the form of a Boltzmann entropy *S* = log Ω, where Ω is the maximum volume occupied by the isolated system. The quantity *ℓ^D^* corresponds to the volume occupied by the random walker. Whenever *ℓ* could reach the maximum size *L* of the sequence, the second term on the right side would write log *L^D^*. The term *ℓ*/*n* in equation (5) represents the excess entropy *S_ext_* introduced by the partition process. It comes into play when the sequence is partitioned in clusters and depends on *n*.

**Figure 2:**
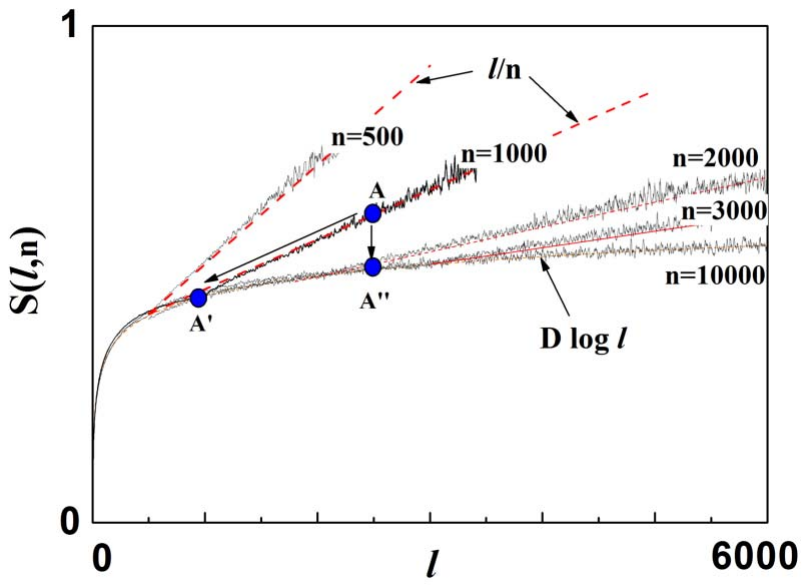
Cluster Entropy. Entropy *S*(*ℓ*, *n*) of the clusters corresponding to the probability distribution function *P*(*ℓ*, *n*) plotted in Fig. 1. For small values of *ℓ*, the curves increase logarithmically as log *ℓ^D^* and are *n*-invariant, while they vary as a linear function for larger values of *ℓ*, as expected according to equation (5).

Fig. 2 shows the entropy *S*(*ℓ*, *n*) evaluated by using the probability distribution *P*(*ℓ*, *n*) plotted in Fig. 1. One can note that *S*(*ℓ*, *n*) increases logarithmically as log *ℓ^D^* and is *n*-invariant for small values of *ℓ*, while it increases as a linear function at larger *ℓ*, as expected according to equation (5). Clusters with lengths *ℓ* larger than *n* are not power-law correlated, due to the finite-size effects introduced by the window *n*. Hence, they are characterized by a value of the entropy exceeding the curve log *ℓ^D^*, which corresponds to powerlaw correlated clusters. It is worthy to remark that clusters with a given length *ℓ* can be generated by different values of the window *n*. For example, clusters with *ℓ* = 2500 have entropies corresponding to the point *A* (for *n* = 1000) or *A*″ (for *n* = 3000 and *n* = 10000) as shown in Fig. 2. One can observe that *A*″ corresponds to power-law correlated (ordered) clusters, since *A*″ lies on the curve log *ℓ^D^*. Conversely, the point *A* does not correspond to power-law correlated clusters, since *A* lies on the curve *ℓ*/*n* which originates from the term 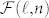. In other words, clusters with lengths shorter than *n* are ordered (long-range correlated), whereas clusters with lengths larger than *n* are disordered (exponentially correlated).

To gain further insight in the meaning of the terms appearing in equation (5), the *source entropy rate s* is calculated for the entropy *S*(*ℓ*, *n*). The source entropy rate is a measure of the *excess randomness* and increases as the block coding process becomes noisier. By using the definition and equation (5), the source entropy rate writes: 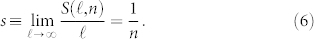
The excess randomness of the clusters is found to be inversely proportional to *n* and, thus, becomes negligible in the limit of *n* → ∞. This clearly occurs in the curves of Fig. 2, where one can note that higher entropy rates correspond to steeper slopes of the linear term *ℓ*/*n* (smaller *n* values).

## Discussion

In this section, the information measure is implemented on the 24 human chromosomes, mapped to fractional Brownian walks (mapping details are described in Method). The nucleotide composition of the DNA sequence is taken as the relevant information quantity to be encoded from the source and decoded from the receiver.

It is well-established that the two strands of DNA are held together by hydrogen bonds between complementary bases: two bonds for the AT pair and three bonds for the GC pair, which is therefore stronger. The existence of GC-rich and GC-poor segments may play different roles in biological processes as duplication, segmentation, unzipping^13^,^14^,^15^.

Nonuniformity of nucleotides composition within genomes was revealed several decades ago by thermal melting and gradient centrifugation. On the basis of findings concerning buoyant densities of melted DNA fragments, a theory for the structure of genomes of warm-blooded vertebrates known as the *isochores theory* was put forward^16^,^17^,^18^,^19^. Isochores were defined as genomic segments that are fairly homogeneous in their guanine and cytosine (GC) composition.

Though it is widely accepted that the human genome contains large regions of distinctive GC content, the availability of fully sequenced DNA or RNA molecules allows one to accurately investigate the local structure by statistical methods. The development of efficient algorithms achieving deep and accurate description of the complex genomic architecture is thus a timely endeavour^20^,^21^,^22^,^23^,^24^,^25^,^26^,^27^,^28^,^29^,^30^.

**Figure 3:**
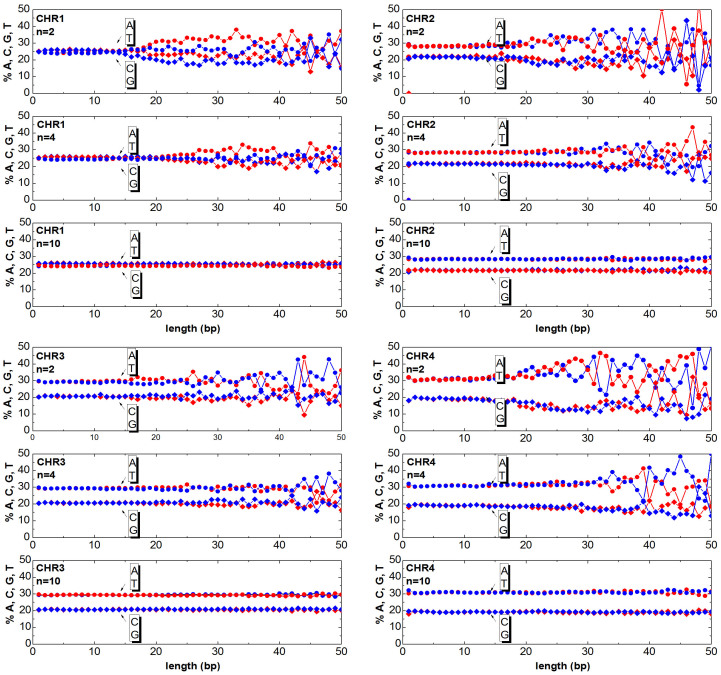
Cluster Composition. Base composition (% of A (blue) T (red) C (blue) G (red) nucleotides) of the clusters in the human chromosomes 1, 2, 3, 4. For each chromosome, the plots refer to windows *n* = 2, *n* = 4, *n* = 10. Data refers to the first 10Mbases of each chromosome. See Tables S1–S6 of Supplementary Information for further estimates.

**Figure 4:**
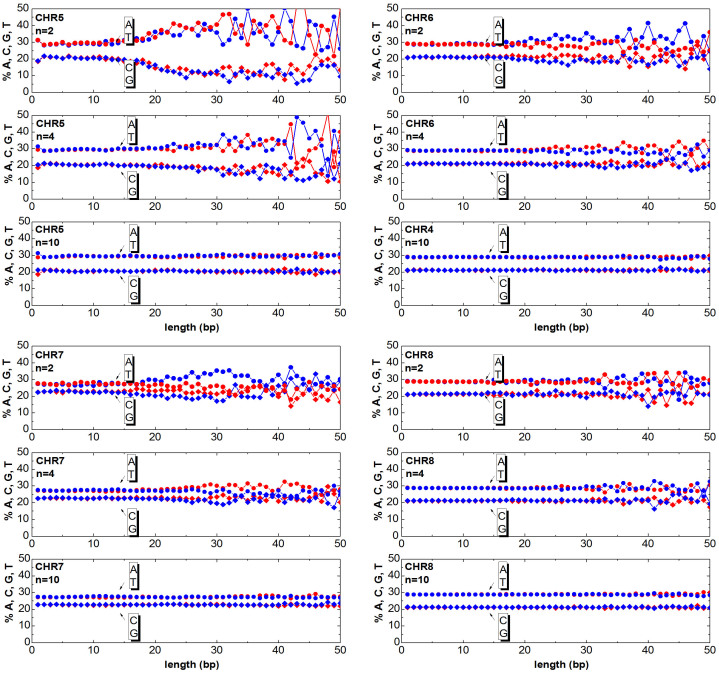
Same as Fig. 3 but for the chromosomes 5, 6, 7, 8.

**Figure 5:**
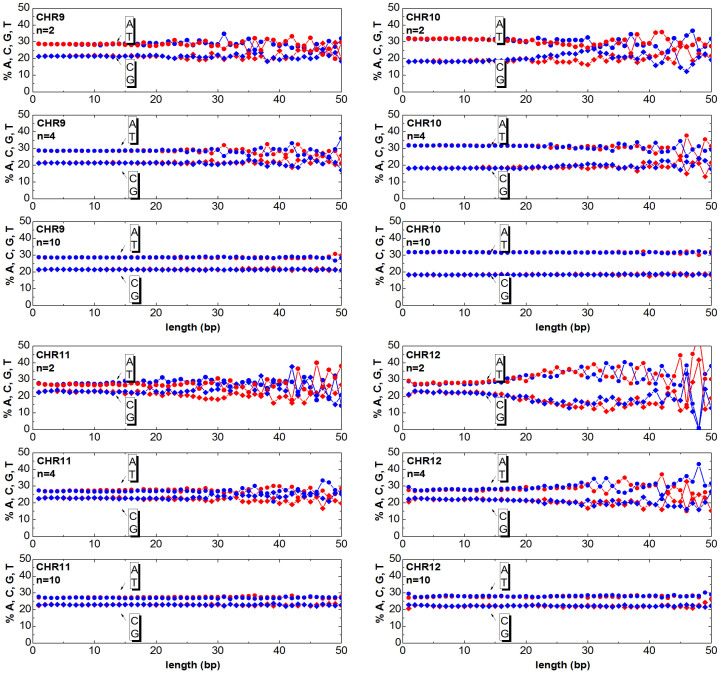
Same as Fig. 3 but for the chromosomes 9, 10, 11, 12.

**Figure 6:**
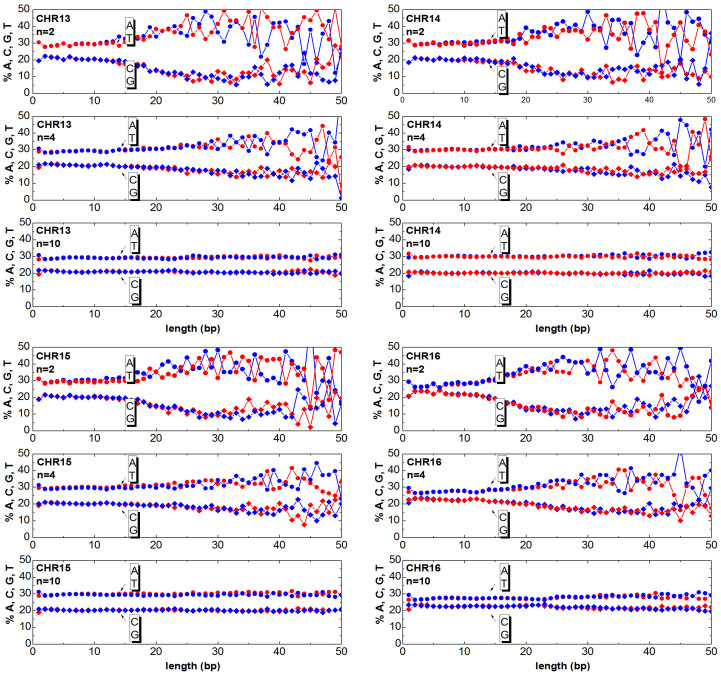
Same as Fig. 3 but for the chromosomes 13, 14, 15, 16.

**Figure 7:**
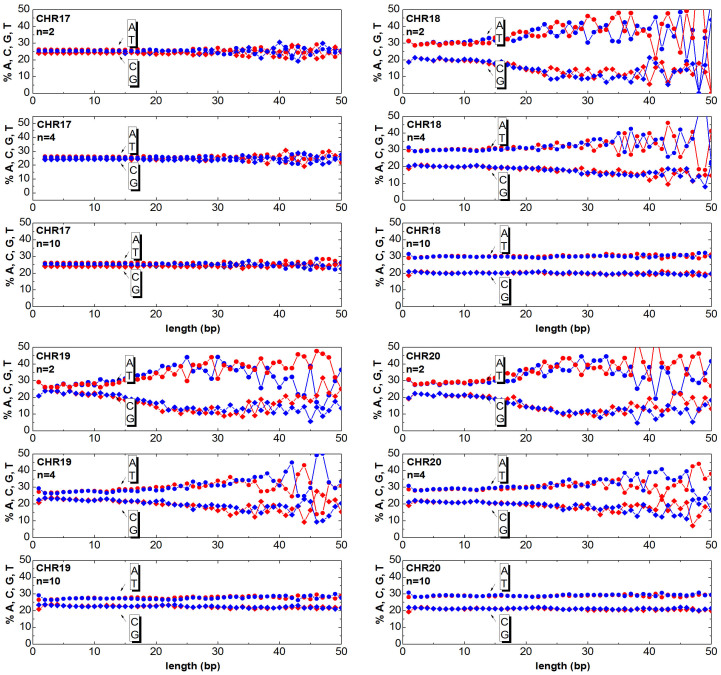
Same as Fig. 3 but for the chromosomes 17, 18, 19, 20.

**Figure 8:**
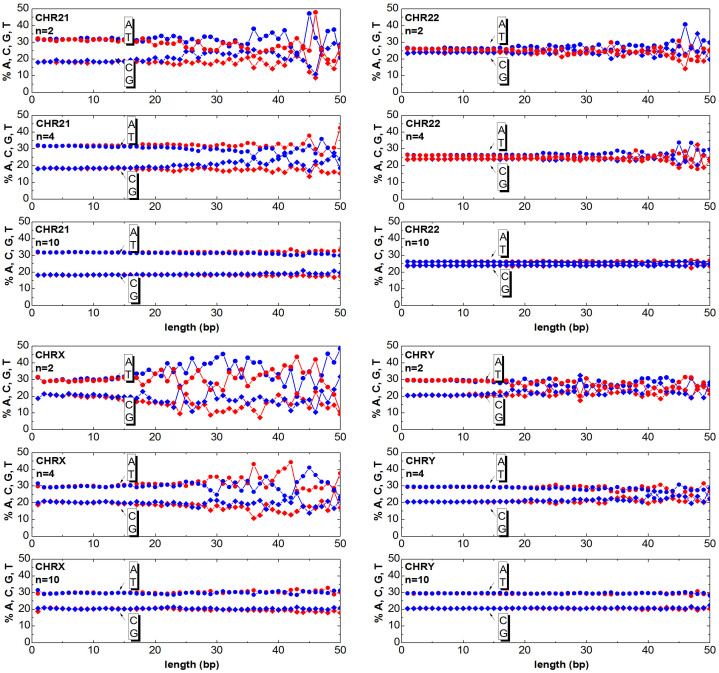
Same as Fig. 3 but for the chromosomes 21, 22, X, Y.

The chromosomes can be mapped to numeric sequences according to different approaches. In this work, first the DNA is mapped (as detailed in the section Method) to a random walk, then the clusters are generated as described in the previous section. Once having generated the clusters, one can answer the question ‘How much of the relevant information is still contained in the clusters?’. The answer to this question is obtained by counting the ATGC basis for each cluster and plotting the percentage as a function of the cluster length. In Figs. 3,4,5,6,7,8, the nucleotide compositions are plotted as a function of the cluster length *ℓ* for *n* = 2, *n* = 4 and *n* = 10. The range of *n* values used in this work varied from 2 to 10.000. One can observe that the nucleotides count is roughly constant for clusters having length comparable or shorter than *n*. This means that *ordered* DNA clusters with constant nucleotide composition are found, when the entropy varies as a logarithm of *ℓ*. For cluster lengths *ℓ* larger than *n*, the power-law correlation breaks down with the onset of exponentially correlated clusters (‘disordered’ clusters). An even more interesting result is that the amplitude of the fluctuations is not constant as it takes a characteristic value for each chromosome. One can note from the data plotted in Figs. 3,4,5,6,7,8 that the fluctuations of the cluster composition is very small for example in chromosomes 8, 9, 17, Y. Conversely, they are quite large for chromosomes 14, 15, X. It should be remarked that Figs. 3,4,5,6,7,8 show the nucleotide composition of the ordered-disordered clusters. These plots are related to the entropy of the blocks if one bears in mind the original aim of the Shannon work. The estimate of the block entropy was originally motivated by the attempt at decoding information in ways that still allow recovery of the relevant information by the receiver. In other words, the main question raised by Claude Shannon is: “How the signal can be compressed in elementary messages (blocks) which still contain the relevant information to be communicated?”. The approach proposed in this work answers this question. The DNA sequence is encoded in short messages (clusters) able to transmit the same information of the whole sequence (from where they were cut out) only if they are power-law correlated. In this manuscript, the information considered relevant to the receiver is the nucleotide composition, which, of course, is not the only choice for the relevant information to be transmitted, as other characteristic features might be interesting as well. It is also discussed to what extent nucleotide fluctuations, characterizing the exponentially correlated clusters of each chromosome, might be linked to features relevant to biological processes. To this purpose, the standard deviation of the fluctuations has been calculated for the nucleotide composition ATGC of the clusters (values are reported in Table 2). The correlation *σ_C_* with bilogical features characteristic of each chromosome, such as length, gene density, inter-chromosomal duplications, intra-chromosomal duplications, local ATGC composition (data taken from Refs. 14, 15) have been considered. The correlation coefficients *ρ_C_* are shown in Table 3. Negative correlations between *σ_C_* and intra-and inter-chromosomal duplications are found. Conversely, strong positive correlations are observed between *σ_C_* and AT-rich regions. These findings might point to the important result that the cluster fluctuations are fingerprints of recent segmental duplications.

## Methods

**Figure 9:**
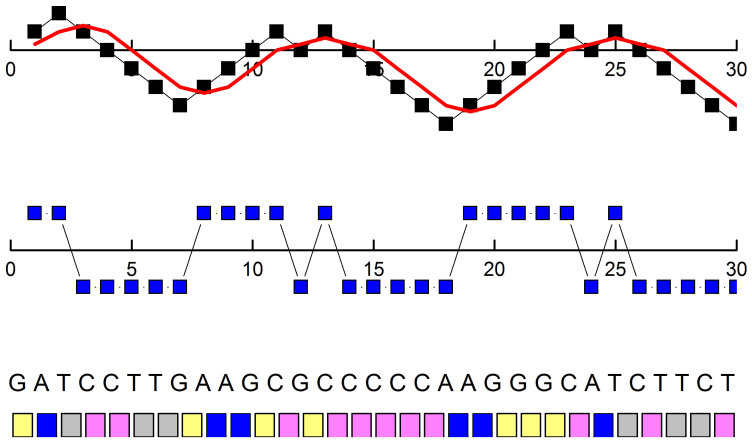
DNA Sequence Mapping Visualization. Bottom: scheme of the first 30 ATGC bases of the sequence of the human chromosome 1. Middle: the sequence of +1 and −1 corresponding to the ATGC. Top: the DNA walk *y*(*x*) obtained by summing the sequence of +1 and −1 (black squares) with the moving average 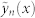 with *n* = 3 (red curve).

A DNA sequence is composed of four nucleotides: adenine (A), thymine (T), cytosine (C) and guanine (G). The first step of the analysis consists in the conversion of the four-letter genome alphabet into a numerical format. There are several ways of mapping a DNA sequence to a walk: one-dimensional up to 4 dimensional, real or complex representations. As the proposed Shannon entropy measure applies to one-dimensional sequences, the present discussion is limited to one-dimensional real representation of the four nucleotide bases. The sequence of the nucleotide bases is mapped according to the following rule: if the base is a purine (A,G), the base is mapped to +1, otherwise if the base is a pyrimidine (C,T), the base is mapped to –1 (Fig. 9). The sequence of +1 and –1 is summed and a random walk *y*(*x*) (*DNA walk*) is obtained. This coding rule is preferable, as it keeps the nonstationarity of the series at a minimum. Large nonstationarity of the numerical series might be an issue when long-range correlation should be investigated. The average concentration of A and T are about 0.30, those of G and C are about 0.20. The concentration of purines (A + G) and pyrimidines (C + T) are very close to 0.50 along the sequence. Therefore, coding of purines and pyrimidines to +1 and −1 guarantees a high degree of symmetry of the numerical series. Conversely, an asymmetric coding rule would amplify the strong variations of the local density distribution of the bases along the sequences, giving rise to higher nonstationarity of the corresponding random walk.

The function 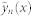 is calculated for the *DNA walk* with different values of the window *n*. The intersection between *y*(*x*) and 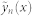 yields a set of clusters, which correspond to the segments between two adjacent intersections of *y*(*x*) and 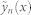. Since each cluster of the *DNA walk* corresponds to a cluster of ATGC nucleotides, the number of nucleotides can be counted and plotted as a function of the length *ℓ* for each cluster. In Figs. 3,4,5,6,7,8 the nucleotide composition of the clusters as a function of the length *ℓ* is shown for the 24 human chromosomes. The clusters have been cut out of 10^6^ bases of each chromosome at once. To be statistically meaningful, there is a need to operate over subsequences having the same length (note that the 24 human chromosomes have different lengths *L*, 2nd column of Table 1). The method proposed here has been however implemented on several sequences with different lengths (varying from 10^5^ to 10^7^ have been considered in this study). This range takes into account that, on one hand, a scaling law is sound when it is observed at least over three decades of a logarithmic scales, and the computational time and complexity on the other hand. One can note that the average composition of the power-law correlated clusters is comparable with the composition of the whole sequence of the analysed data. For example the nucleotide composition of the power-law correlated clusters of the chromosome 1 should be confronted with the data reported in the column 8*^th^*, 9*^th^*, 10*^th^*, 11*^th^* of Table 1 for the same chromosome, while the standard deviation is reported in Table 2. The statistical robustness of the method has been checked by estimating the correlation coefficient *ρ_c_* of the variance and other biological parameters of the sequences (Table 3).

One common problem in data mining is the statistical validation of the model envisioned to describe data structures and patterns. The error is estimated on the entire sample set for small quantity of data. For large data sets, more sophisticated cross-validation methods have been developed to quantify the performance of algorithms and models over disjoint subsets. Depending upon the criterion used to split the data, the process of training and validation across disjoint sets is named *random*, *k-fold* or *leave-one-out*^31^. In particular, the leave-one-out is the degenerate case of the *k*-fold cross-validation, with only one disjoint subset (*k* = 1) and is particularly useful for very sparse datasets with few samples, though its error might be larger than the error of the estimates themselves and computation time might be quite long. As the analysed dataset (the 24 genomic sequences) is large enough, the random and *k*-fold cross validation can be used with the advantage of higher accuracy and velocity of the estimates. In the Supplementary Tables S1–S6, the average values and variances of the nucleotide contents obtained over three disjoint data sets are reported for the 24 chromosomes. For each subset, when the parameter *n* is varied, clusters of any lengths are generated in random position of the sequence allowing to estimate the average composition and the statistical errors at different position along the sequence. For each set the standard deviations are also reported in the Supplementary Tables S1–S6.

Finally, we note that the Hurst exponent for the 24 chromosomes is reported in the 3*^rd^* column of Table 1. As one can see the value of the exponent *H* is higher than 0.5, implying that a positive correlation (persistence) exist among the nucleotides. The values of the Hurst exponents have been obtained by using the method described in Refs. 8,9,10.

The sequences used in this analysis were retrieved from the NCBI ftp server (ftp://ftp.ncbi.nlm.nih.gov/genomes/H_sapiens/).

## Supplementary Material

Supplementary Information Information measure for long range correlated sequence

## Figures and Tables

**Table 1:** Nucleotide Composition. Length *L* (2*^nd^* column), Hurst exponent *H* (3*^rd^* column), base composition (% of ATCG, 4*^th^*–7*^th^* columns) of the 24 chromosome whole sequences. Average nucleotide composition (% of the ATCG, 8*^th^*–11*^th^* columns) of the clusters, estimated according to the proposed method with *n* = 4 over the first 10MBases of the 24 chromosome sequences. In particular, the data in the 8*^th^*–11*^th^* columns correspond to the plots shown in the middle panels of Figs. 3,4,5,6,7,8 for each chromosome. In Tables S1–S6 of Supplementary Information, further results, estimated over different data sets with different values of *n*, are reported

CHR	*L*	*H*	A [%]	C[%]	G[%]	T[%]	A[%]	C[%]	G[%]	T[%]
1	226217758	0.64	29.09	20.87	20.87	29.14	26.52	25.79	25.58	25.15
2	237900011	0.66	29.84	20.11	20.13	29.90	28.50	24.51	22.34	29.81
3	195304882	0.66	30.14	19.84	19.84	30.16	28.46	21.77	21.50	28.65
4	187941502	0.66	30.87	19.11	19.12	30.88	34.47	19.80	22.86	30.28
5	177847050	0.66	30.20	19.74	19.77	30.27	29.97	24.86	19.70	36.43
6	169100547	0.65	30.18	19.80	19.81	30.19	29.97	21.23	21.73	28.27
7	155403473	0.66	29.60	20.38	20.36	29.63	28.85	21.79	27.09	22.93
8	143332430	0.65	29.90	20.06	20.06	29.86	29.51	20.61	20.85	29.03
9	120994158	0.67	29.35	20.65	20.64	29.33	27.91	21.83	21.38	28.76
10	131739836	0.65	29.19	20.79	20.78	29.22	31.15	19.94	19.47	29.44
11	131247160	0.68	29.20	20.77	20.79	29.21	28.97	22.23	25.08	26.85
12	130304143	0.67	29.59	20.40	20.39	29.60	30.66	22.85	24.19	29.62
13	95747346	0.66	30.69	19.26	19.26	30.77	33.94	20.95	21.82	33.88
14	88290585	0.67	29.44	20.41	20.46	29.67	33.94	20.95	21.82	33.88
15	81927784	0.66	28.89	21.13	21.10	28.86	32.64	21.69	20.74	33.00
16	78990748	0.67	27.53	22.35	22.44	27.66	29.17	24.30	22.89	31.49
17	79620483	0.65	27.17	22.81	22.76	27.22	25.36	24.80	24.73	24.87
18	74660927	0.67	30.09	19.87	19.90	30.12	25.36	24.80	24.73	24.87
19	56038018	0.66	25.79	24.14	24.20	25.86	32.65	21.61	23.15	30.64
20	59505758	0.66	27.76	22.02	22.09	28.10	29.01	24.09	19.02	36.45
21	35452914	0.65	29.68	20.39	20.44	29.46	32.27	19.25	21.18	27.29
22	35059666	0.65	26.08	23.98	23.95	25.96	28.30	22.93	24.63	24.92
X	152580014	0.65	30.20	19.73	19.76	30.26	32.88	20.86	25.39	28.01
Y	25654723	0.72	29.88	19.87	20.08	30.14	27.45	22.05	24.21	26.49

**Table 2:** Standard deviation of the cluster nucleotide composition. Standard deviations refer to the average values (% of the ATCG, 8*^th^*–11*^th^* columns), estimated according to the proposed method with *n* = 4 over the first 10MBases of the 24 chromosome sequences. Standard deviationscan be appreciated in the middle panel plots of Figs. 3,4,5,6,7,8 for each chromosome. In Tables S1–S6 of Supplementary Information, further values over different chromosome sets and with different values of *n* are reported

CHR	*σ_C_* [A]	*σ_C_* [C]	*σ_C_* [G]	*σ_C_* [T]
1	11.01	10.81	10.06	9.68
2	14.19	12.90	12.43	14.30
3	10.05	9.43	8.29	8.52
4	16.56	12.91	13.87	17.79
5	19.75	14.32	12.76	16.69
6	9.07	6.33	7.42	8.71
7	8.58	8.32	11.14	10.21
8	4.89	3.88	4.33	4.91
9	6.49	4.97	4.36	5.23
10	10.84	9.04	7.52	9.38
11	9.12	8.83	11.69	10.87
12	17.09	14.86	14.13	15.63
13	17.12	12.01	13.78	18.11
14	19.53	13.06	13.43	19.26
15	16.55	14.08	12.54	16.61
16	16.31	15.60	15.77	15.69
17	5.06	5.17	4.95	5.67
18	20.02	13.98	14.10	18.47
19	17.90	14.88	13.99	17.10
20	17.31	13.86	14.33	18.70
21	10.84	7.69	8.50	10.81
22	8.40	5.81	5.53	8.48
X	18.06	14.09	14.87	19.06
Y	6.19	6.66	7.08	7.47

**Table 3:** Correlation *ρ_C_* of the cluster fluctuations for the first (M_1_), the second (M_2_) and the third (M_3_) disjoint sets of the 24 human chromosome sequences. The fluctuations are anticorrelated with length, gene density, inter-chromosomal and intra-chromosomal segmental duplications, while they exhibit a positive correlation with the AT-rich regions. Very little correlation is found with the GC-rich regions and global AT composition. Length values are shown in the 2*^nd^* column of Table 1. Gene density data are taken from Refs. 14, 15. Inter- and intra-chromosomal duplications data are taken from Ref. 14. Base compositions are shown in Table 1 (respectively 4*^th^*–7*^th^* columns for the whole sequence, 8*^th^*–11*^th^* columns for the first 10MBases, and in Tables S1–S6 of the Supplementary Information)

	*ρ_C_* [M_1_]	*ρ_C_* [M_2_]	*ρ_C_* [M_3_]
Length	−0.194	−0.552	−0.582
Gene density	−0.178	−0.076	−0.107
Inter-chromosomal duplications	−0.330	−0.242	−0.165
Intra-chromosomal duplications	−0.342	−0.248	−0.158
All pairwise duplications	−0.331	−0.237	−0.149
Local composition A	+0.658	+0.762	+0.461
Local composition T	+0.668	+0.674	+0.551
Local composition C	+0.021	+0.039	+0.269
Local composition G	−0.149	+0.211	+0.246
Global composition AT	+0.052	−0.154	−0.219
