# Supplementary material for: Information Measure for Long-Range Correlated Sequences: the Case of the 24 Human Chromosomes
Source: Sci Rep. 2013 Sep 23;3:2721. doi: 10.1038/srep02721 (PMC3779848; doi:10.1038/srep02721)
Supplement: Supplementary Information — Information measure for long range correlated sequence [file srep02721-s1.pdf]

Information Measure for Long-Range Correlated Sequences: the case of the 24 Human Chromosomes. Supplementary Information

A. Carbone[1,2,3,*]

August 22, 2013

[1] *Politecnico di Torino, Italy*
[2] *ISC-CNR, Unità Università 'La Sapienza' di Roma, Italy*
[3] *ETH Zurich, Switzerland*

The average values and variance of the nucleotide contents obtained over three disjoint data sets is shown for the 24 chromosomes in Supplementary Tables 1-8. For each set, the parameter n has been varied in order to generate clusters of any lengths in random position of the sequence. The standard deviations are also reported in the same tables.

Further data, statistics and all the codes can be downloaded at the website `www.polito.it/noiselab` in the section *Utilities*.

	CHROMOSOME 1									CHROMOSOME 2								
	n	A [%]	σ_C [A]	C[%]	σ_C [C]	G[%]	σ_C [G]	T[%]	σ_C [T]	n	A [%]	σ_C [A]	C[%]	σ_C [C]	G[%]	σ_C [G]	T[%]	σ_C [T]
$k=1$	2	27.31	10.11	25.24	11.34	24.81	9.95	25.58	8.29	2	29.87	12.57	24.86	11.62	23.01	11.45	32.68	12.56
	4	26.52	11.01	25.79	10.81	25.58	10.06	25.15	9.68	4	28.50	14.19	24.51	12.90	22.34	12.43	29.81	14.30
	6	24.31	9.62	26.05	10.93	25.61	9.83	24.72	9.53	6	26.46	13.42	24.43	12.80	22.25	12.77	29.01	13.16
	8	24.68	10.11	25.95	11.33	26.17	10.65	23.59	9.55	8	27.23	12.09	23.01	11.74	22.22	12.09	28.13	12.36
	10	24.48	8.96	25.89	10.46	26.18	10.27	23.77	8.78	10	26.10	10.89	23.63	11.12	22.55	10.94	28.25	11.01
$k=2$	2	25.94	4.95	23.64	4.50	24.24	5.45	26.18	5.79	2	27.81	5.29	21.76	4.57	23.40	5.81	27.02	6.36
	4	25.47	6.02	24.08	5.25	24.21	5.99	26.56	7.04	4	27.57	5.45	21.76	5.12	23.07	5.65	27.59	7.19
	6	25.92	5.66	23.78	5.12	24.49	6.17	25.95	6.25	6	28.08	5.84	21.86	4.92	22.94	5.47	27.10	6.30
	8	25.88	5.38	23.80	4.89	24.51	5.41	25.81	5.39	8	28.04	5.38	21.84	4.70	23.04	5.80	27.06	5.84
	10	25.82	5.22	24.04	4.81	24.66	5.39	25.48	5.33	10	27.94	5.29	22.03	4.80	22.80	4.79	27.21	5.27
$k=3$	2	26.62	6.09	23.00	5.32	25.04	6.18	25.84	5.82	2	30.50	4.45	20.14	4.42	20.16	4.12	29.19	4.72
	4	26.33	6.14	23.26	4.75	24.85	5.66	25.69	6.10	4	30.52	4.97	19.94	4.07	19.63	3.92	29.92	5.23
	6	26.07	5.41	23.67	4.94	24.67	5.27	25.58	5.33	6	30.29	4.82	19.84	3.99	19.68	3.74	30.18	4.87
	8	25.84	5.64	24.44	5.67	24.64	5.45	25.34	5.26	8	30.50	3.82	19.88	3.81	19.54	3.21	30.08	4.28
	10	25.90	5.01	23.91	4.85	25.09	4.99	25.10	4.71	10	30.31	3.94	19.77	3.58	19.42	3.60	30.49	4.44

	CHROMOSOME 3									CHROMOSOME 4								
	n	A [%]	σ_C [A]	C[%]	σ_C [C]	G[%]	σ_C [G]	T[%]	σ_C [T]	n	A [%]	σ_C [A]	C[%]	σ_C [C]	G[%]	σ_C [G]	T[%]	σ_C [T]
$k=1$	2	29.32	10.13	22.17	9.85	22.44	8.30	28.32	8.32	2	39.10	15.15	22.24	13.55	24.20	13.21	35.90	15.69
	4	28.46	10.05	21.77	9.43	21.50	8.29	28.65	8.52	4	34.47	16.56	19.80	12.91	22.86	13.87	30.28	17.79
	6	28.72	9.70	21.61	8.85	21.93	8.38	28.08	8.51	6	31.74	17.41	19.41	12.92	21.92	13.76	29.21	18.12
	8	28.59	9.27	21.35	8.42	22.15	8.37	28.08	8.55	8	31.59	16.35	18.64	11.49	21.69	13.52	29.15	17.90
	10	28.49	9.01	22.00	8.91	22.17	8.45	27.62	8.06	10	30.98	15.47	18.92	11.16	20.91	12.18	29.28	15.90
$k=2$	2	28.68	6.90	20.84	4.68	23.39	7.00	27.46	8.54	2	30.13	9.37	19.75	6.39	19.27	7.70	30.85	9.89
	4	28.43	5.62	21.10	4.64	23.13	5.90	27.73	6.59	4	31.20	8.37	18.93	5.45	18.72	6.98	31.30	9.67
	6	27.76	6.23	21.54	4.73	22.86	5.96	28.03	6.77	6	31.30	8.21	18.94	6.12	18.28	5.78	31.48	8.66
	8	27.07	5.20	22.35	4.58	22.64	5.18	27.95	6.45	8	31.20	8.13	18.89	5.41	17.94	6.19	31.96	9.04
	10	27.39	5.14	22.46	5.20	23.03	5.02	27.12	5.56	10	31.10	8.14	18.81	5.00	18.12	5.84	31.97	9.13
$k=3$	2	30.73	6.31	19.21	4.50	19.99	4.27	30.37	5.37	2	31.09	5.36	19.86	5.24	20.44	6.64	29.08	6.77
	4	31.25	5.96	18.87	4.17	19.48	3.64	30.65	4.90	4	30.77	5.61	19.27	4.96	20.04	5.88	29.93	6.42
	6	30.67	4.88	19.29	4.02	19.57	4.32	30.47	5.21	6	31.17	5.90	19.24	4.72	20.10	5.11	29.49	6.37
	8	30.84	4.35	19.04	3.27	19.56	3.98	30.56	5.85	8	31.42	5.57	19.08	4.11	20.04	5.22	29.46	5.89
	10	30.12	5.09	19.46	3.70	19.94	4.20	30.49	5.11	10	31.25	5.16	19.21	4.25	20.01	4.82	29.53	5.89

Table 1: Average Nucleotide Composition of the Clusters. Base composition and standard deviation of nucleotide % of A (3rd and 4th columns), % of C (5th and 6th columns), % of G (7th and 8th columns), % of T (9th and 10th columns). Different $k's$ refer to the first (M_1), second (M_2) and third (M_3) disjoint sets of the chromosomes 1, 2, 3, 4. Different values of n are shown also.

	CHROMOSOME 5									CHROMOSOME 6								
	n	A [%]	σ_C [A]	C[%]	σ_C [C]	G[%]	σ_C [G]	T[%]	σ_C [T]	n	A [%]	σ_C [A]	C[%]	σ_C [C]	G[%]	σ_C [G]	T[%]	σ_C [T]
$k=1$	2	38.67	16.10	26.08	13.44	20.28	11.83	42.62	13.40	2	30.20	9.93	21.79	7.09	21.10	6.63	28.49	8.97
	4	29.97	19.75	24.86	14.32	19.70	12.76	36.43	16.69	4	29.97	9.07	21.23	6.33	21.73	7.42	28.27	8.71
	6	27.90	19.23	24.21	13.69	19.11	11.78	33.24	17.76	6	29.00	8.84	22.04	7.86	21.67	7.58	27.75	8.37
	8	26.77	16.82	24.07	12.89	18.83	11.45	32.46	16.22	8	28.69	8.83	22.15	8.10	21.44	6.50	28.30	8.17
	10	26.72	16.00	23.66	12.64	19.19	11.29	31.19	15.47	10	28.35	7.90	22.54	7.63	21.03	7.12	28.33	7.60
$k=2$	2	34.90	16.10	21.89	13.00	23.40	10.37	33.17	15.08	2	28.77	5.30	21.47	4.72	21.11	3.97	28.65	5.46
	4	32.36	16.35	21.60	12.87	23.98	12.58	30.32	16.83	4	28.59	5.14	21.33	4.26	21.46	3.85	28.62	5.13
	6	28.81	15.12	22.37	12.87	21.98	11.66	29.16	16.07	6	28.45	5.77	21.66	5.25	21.47	4.44	28.38	5.03
	8	29.67	14.59	21.70	12.20	22.39	11.59	27.21	14.74	8	28.48	5.60	21.36	5.65	21.43	3.79	28.67	5.06
	10	29.18	12.77	21.27	10.16	21.99	9.79	28.06	13.01	10	28.64	5.11	21.20	3.88	21.21	3.64	28.85	4.77
$k=3$	2	30.18	10.32	19.67	8.34	23.38	9.83	28.88	12.60	2	30.73	5.02	19.02	4.47	19.94	4.74	30.31	6.04
	4	30.94	11.06	18.89	7.70	23.18	10.12	28.08	12.35	4	30.67	4.97	19.47	4.60	19.99	4.22	29.87	6.10
	6	30.71	10.08	19.16	7.18	22.21	9.14	28.06	11.45	6	30.33	4.60	19.85	4.31	20.26	3.60	29.55	5.22
	8	30.69	9.52	18.75	6.80	21.84	9.15	28.89	11.13	8	30.39	4.59	19.59	4.03	20.05	3.67	29.97	4.97
	10	30.44	9.13	18.89	6.49	21.70	8.72	29.13	10.66	10	30.50	4.64	19.64	3.84	20.13	4.30	29.73	5.36
	CHROMOSOME 7									CHROMOSOME 8								
	n	A [%]	σ_C [A]	C[%]	σ_C [C]	G[%]	σ_C [G]	T[%]	σ_C [T]	n	A [%]	σ_C [A]	C[%]	σ_C [C]	G[%]	σ_C [G]	T[%]	σ_C [T]
$k=1$	2	29.06	8.12	21.65	8.09	26.51	10.55	23.22	9.51	2	29.46	4.78	20.44	4.08	21.30	4.23	28.79	4.65
	4	28.85	8.58	21.79	8.32	27.09	11.14	22.93	10.21	4	29.51	4.89	20.61	3.88	20.85	4.33	29.03	4.91
	6	27.51	8.34	22.31	8.85	26.65	10.31	23.65	9.47	6	29.18	4.85	20.85	3.88	21.23	4.10	28.74	4.45
	8	26.88	8.22	23.33	10.23	25.85	10.49	24.07	8.90	8	28.74	5.21	21.17	4.22	21.94	4.97	28.15	4.72
	10	26.38	7.91	23.97	9.78	25.28	9.83	24.59	8.17	10	28.46	4.67	20.91	3.88	22.21	4.88	28.43	4.45
$k=2$	2	32.16	8.29	19.75	6.25	19.37	7.27	29.04	8.29	2	30.33	5.23	20.37	5.01	21.02	4.78	28.28	5.36
	4	31.99	6.91	18.97	4.99	19.38	6.41	29.94	7.62	4	30.44	4.66	20.30	4.35	20.65	5.24	28.61	4.99
	6	31.87	6.03	18.93	4.83	18.84	5.51	30.49	6.76	6	30.28	4.74	20.53	4.66	20.44	4.32	28.75	4.90
	8	31.43	5.29	18.76	4.20	18.65	5.27	31.28	6.69	8	30.43	4.68	20.29	4.38	20.40	4.41	28.88	5.23
	10	31.40	5.45	18.70	4.13	18.59	4.47	31.31	6.11	10	30.15	4.88	20.36	4.63	20.52	4.80	28.97	4.51
$k=3$	2	30.60	6.82	20.99	4.29	19.99	4.94	28.43	6.46	2	27.97	6.08	22.15	4.99	21.72	4.42	28.16	6.32
	4	29.28	7.22	21.43	4.72	20.28	5.10	29.01	7.62	4	27.87	5.33	22.38	5.20	21.63	4.41	28.12	5.59
	6	29.19	5.73	21.07	4.05	20.73	4.90	29.01	6.28	6	28.00	4.78	21.75	4.48	22.08	4.14	28.18	5.45
	8	29.57	5.63	20.86	3.96	20.67	4.94	28.91	6.07	8	28.27	4.71	21.95	4.26	21.73	4.21	28.06	5.15
	10	29.48	5.45	20.67	3.97	20.70	4.45	29.15	5.68	10	27.69	4.63	22.01	4.31	22.13	4.04	28.17	4.90

Table 2: Same as Table 1 but for the chromosomes 5, 6, 7, 8.

	CHROMOSOME 9									CHROMOSOME 10								
	n	A [%]	σ_C [A]	C[%]	σ_C [C]	G[%]	σ_C [G]	T[%]	σ_C [T]	n	A [%]	σ_C [A]	C[%]	σ_C [C]	G[%]	σ_C [G]	T[%]	σ_C [T]
	2	28.47	5.05	21.72	4.61	20.93	4.92	28.87	5.52	2	31.61	9.77	19.73	8.05	19.88	7.08	29.08	8.92
	4	27.91	6.49	21.83	4.97	21.38	4.36	28.76	5.23	4	31.15	10.84	19.94	9.04	19.47	7.52	29.44	9.38
$k=1$	6	27.84	6.07	22.07	6.37	21.94	5.03	28.21	5.71	6	31.59	9.73	20.05	7.63	19.56	8.08	29.46	10.13
	8	27.99	6.19	22.19	7.00	21.46	5.65	28.29	5.17	8	30.85	10.08	20.34	8.58	18.82	7.72	30.12	9.73
	10	27.96	5.03	22.05	6.37	21.89	5.59	28.04	4.92	10	31.08	9.55	20.40	8.43	18.10	7.38	30.53	8.92
	2	29.60	5.12	20.23	4.27	20.20	4.67	29.98	6.23	2	29.87	11.56	19.91	7.52	24.02	8.49	27.02	11.13
	4	29.36	6.14	20.75	4.63	19.51	4.61	30.38	6.14	4	30.28	11.01	19.59	7.60	22.90	8.37	27.98	10.54
$k=2$	6	29.55	4.68	20.19	3.94	19.80	4.24	30.45	5.58	6	30.38	10.44	19.42	7.48	21.99	8.48	29.04	10.80
	8	29.68	4.73	20.13	3.81	19.73	3.69	30.46	4.79	8	30.99	9.40	19.22	6.53	21.17	7.71	28.98	9.39
	10	29.47	4.48	20.34	3.77	19.74	3.16	30.45	4.72	10	30.63	8.43	19.68	5.99	21.00	7.43	28.87	8.91
	2	30.03	5.76	20.57	4.51	21.83	5.19	27.78	4.77	2	27.22	9.61	21.98	7.65	24.82	9.16	26.85	11.75
	4	29.85	5.06	20.59	3.97	21.60	4.51	27.96	4.98	4	27.53	9.86	21.86	7.66	24.90	9.57	25.90	11.44
$k=3$	6	29.56	4.86	20.83	3.98	21.58	4.42	28.03	4.81	6	28.31	9.00	21.34	7.05	24.67	8.44	25.85	9.76
	8	29.31	4.58	20.97	3.82	21.50	3.80	28.23	4.47	8	28.18	8.98	22.08	7.09	23.66	7.41	26.24	9.08
	10	29.14	4.69	21.10	3.72	21.55	3.58	28.22	4.00	10	27.46	8.53	22.20	6.99	23.74	8.08	26.59	9.50

	CHROMOSOME 11									CHROMOSOME 12								
	n	A [%]	σ_C [A]	C[%]	σ_C [C]	G[%]	σ_C [G]	T[%]	σ_C [T]	n	A [%]	σ_C [A]	C[%]	σ_C [C]	G[%]	σ_C [G]	T[%]	σ_C [T]
	2	29.26	9.87	21.99	7.82	24.40	11.32	27.47	10.11	2	34.76	14.95	24.49	13.32	25.09	13.05	34.26	13.25
	4	28.97	9.12	22.23	8.83	25.08	11.69	26.85	10.87	4	30.66	17.09	22.85	14.86	24.19	14.13	29.62	15.63
$k=1$	6	26.63	8.87	23.26	9.46	24.78	12.88	26.66	11.31	6	30.02	16.87	21.91	14.19	24.09	14.12	27.59	15.58
	8	26.28	9.23	23.93	10.76	23.93	11.97	26.28	10.33	8	27.79	14.28	23.12	12.79	22.88	13.10	27.62	14.11
	10	25.65	9.03	23.52	10.98	24.83	13.14	26.23	10.48	10	26.73	13.38	23.06	12.73	23.35	13.05	27.37	13.31
	2	27.26	11.01	21.67	7.27	21.50	8.77	30.41	13.20	2	28.06	10.73	20.87	7.88	21.15	9.12	30.17	11.40
	4	27.02	10.15	21.86	8.03	20.83	8.05	30.48	11.75	4	28.45	10.97	20.27	7.68	20.31	9.67	30.97	11.56
$k=2$	6	27.48	10.23	21.61	7.74	20.90	8.36	30.18	11.51	6	28.98	10.18	20.31	7.16	19.92	7.94	31.13	10.87
	8	27.58	9.68	21.78	7.39	21.12	8.18	29.52	11.08	8	29.41	9.46	20.19	6.58	19.80	7.58	30.61	10.08
	10	27.60	9.69	21.96	7.93	21.01	7.43	29.71	10.35	10	29.06	9.23	20.36	6.67	19.83	7.71	30.75	9.65
	2	30.18	11.93	20.84	11.12	21.56	10.35	29.10	11.36	2	29.75	11.94	20.78	8.79	21.48	9.97	29.38	13.11
	4	30.14	11.36	20.66	10.53	20.14	9.56	29.49	11.32	4	29.85	10.84	20.83	8.62	20.24	8.97	30.15	12.35
$k=3$	6	30.55	10.57	20.02	9.98	20.14	9.46	29.60	11.05	6	29.53	10.20	20.62	8.75	19.72	8.66	30.59	11.18
	8	29.78	10.89	20.87	10.23	19.24	9.04	30.23	10.77	8	30.83	9.56	20.07	7.30	19.40	8.59	29.88	10.96
	10	30.02	9.48	20.36	8.70	19.30	8.14	30.32	9.51	10	30.30	9.38	20.05	7.03	19.33	8.09	30.33	10.43

Table 3: Same as Table 1 but for the chromosomes 9, 10, 11, 12.

	CHROMOSOME 13									CHROMOSOME 14								
	n	A [%]	σ_C [A]	C[%]	σ_C [C]	G[%]	σ_C [G]	T[%]	σ_C [T]	n	A [%]	σ_C [A]	C[%]	σ_C [C]	G[%]	σ_C [G]	T[%]	σ_C [T]
	2	40.65	14.23	22.09	12.40	23.04	13.69	41.30	14.70	2	41.94	15.83	20.20	11.03	23.04	13.01	40.63	17.09
	4	33.94	17.12	20.95	12.01	21.82	13.78	33.88	18.11	4	35.84	19.53	20.02	13.06	21.92	13.43	30.95	19.26
$k=1$	6	30.35	16.75	20.83	12.45	21.90	12.49	30.88	17.42	6	34.11	17.06	18.23	11.65	23.30	12.87	27.56	18.13
	8	29.31	15.73	21.22	11.54	21.18	12.66	30.10	16.99	8	32.72	17.23	18.49	11.55	23.28	12.98	27.16	17.71
	10	29.54	14.63	21.12	11.21	21.56	11.51	29.00	15.72	10	31.44	15.34	19.18	11.52	22.63	12.56	27.63	15.97
	2	42.73	17.08	21.84	11.77	22.74	13.27	43.58	15.19	2	40.24	16.10	23.25	14.31	21.61	13.92	40.57	12.29
	4	35.36	19.85	20.15	13.49	20.58	12.48	35.63	17.46	4	33.37	18.24	21.03	13.95	20.33	14.74	34.19	18.21
$k=2$	6	35.00	20.06	18.97	11.70	21.21	12.20	30.10	19.56	6	30.48	19.31	21.66	15.06	19.76	14.53	31.55	18.48
	8	32.55	17.25	18.68	10.58	21.90	11.83	28.91	16.84	8	29.30	16.72	21.21	12.93	19.25	12.89	31.43	16.17
	10	31.63	17.27	19.06	10.87	21.78	10.49	28.56	16.23	10	29.92	15.80	20.79	11.81	19.14	11.12	30.63	14.70
	2	41.87	14.57	20.52	12.37	25.57	14.60	37.41	13.91	2	41.55	15.36	22.11	14.66	23.34	12.15	37.42	12.47
	4	34.21	17.35	19.82	14.46	24.50	15.43	28.82	18.55	4	36.90	18.39	20.44	15.14	22.74	13.00	32.29	17.48
$k=3$	6	32.78	16.31	19.16	13.24	24.29	13.45	27.20	16.99	6	33.79	17.42	19.15	13.34	22.07	12.46	30.30	15.69
	8	32.22	15.61	19.01	12.64	24.26	13.08	26.31	16.03	8	33.86	16.52	18.09	12.17	20.97	11.97	28.82	15.26
	10	30.65	14.53	19.87	11.66	22.75	12.32	27.88	15.07	10	33.24	15.21	18.15	10.94	20.18	10.81	29.37	14.49

	CHROMOSOME 15									CHROMOSOME 16								
	n	A [%]	σ_C [A]	C[%]	σ_C [C]	G[%]	σ_C [G]	T[%]	σ_C [T]	n	A [%]	σ_C [A]	C[%]	σ_C [C]	G[%]	σ_C [G]	T[%]	σ_C [T]
	2	40.84	14.37	22.25	13.19	23.12	13.67	40.13	14.71	2	34.88	13.87	26.22	14.91	23.30	13.63	37.54	14.59
	4	32.64	16.55	21.69	14.08	20.74	12.54	33.00	16.61	4	29.17	16.31	24.30	15.60	22.89	15.77	31.49	15.69
$k=1$	6	28.66	17.57	21.93	13.45	20.68	12.84	30.43	16.80	6	26.91	17.84	24.44	15.90	21.56	15.33	29.44	16.86
	8	27.51	16.52	22.48	13.72	20.39	12.91	30.04	16.31	8	26.43	16.97	23.79	15.97	22.74	15.51	27.78	16.10
	10	28.58	15.42	22.23	12.96	20.69	11.69	29.10	14.67	10	26.67	15.58	23.77	15.40	23.00	14.99	26.56	14.94
	2	40.26	14.52	22.73	16.03	23.13	14.63	36.79	11.11	2	38.33	14.90	24.35	13.47	23.99	12.90	38.60	15.42
	4	32.34	18.19	22.98	16.33	21.29	14.48	31.79	16.12	4	31.94	17.61	22.15	13.18	22.26	13.36	33.44	18.60
$k=2$	6	31.23	17.22	21.30	14.22	22.31	13.14	28.10	16.50	6	29.28	18.50	22.01	14.21	22.22	14.79	30.41	19.57
	8	30.73	15.97	21.10	13.49	21.56	12.23	27.67	14.99	8	27.47	17.88	22.30	13.95	21.70	15.03	29.83	18.42
	10	30.12	14.71	20.70	11.97	21.94	11.71	27.72	14.31	10	26.21	17.27	22.85	14.23	21.21	14.77	30.13	17.77
	2	37.32	10.29	23.57	14.73	21.14	13.91	42.04	15.39	2	33.14	12.52	22.66	10.96	21.99	12.25	34.38	15.95
	4	30.90	18.55	23.68	15.16	19.56	13.97	35.87	18.51	4	28.95	13.80	22.68	11.12	21.02	11.42	31.08	15.86
$k=3$	6	26.78	15.31	22.30	12.87	19.83	14.77	34.00	17.49	6	26.82	13.90	23.25	12.02	20.76	11.06	30.52	14.57
	8	27.56	14.08	21.79	11.45	19.29	11.82	32.83	15.67	8	26.30	13.54	23.29	11.90	20.48	11.38	30.87	14.32
	10	27.57	13.71	21.65	11.93	20.55	13.03	31.30	15.35	10	27.63	12.70	21.72	10.19	21.03	10.32	30.07	13.09

Table 4: Same as Table 1 but for the chromosomes 13, 14, 15, 16.

	CHROMOSOME 17									CHROMOSOME 18								
	n	A [%]	σ_C [A]	C[%]	σ_C [C]	G[%]	σ_C [G]	T[%]	σ_C [T]	n	A [%]	σ_C [A]	C[%]	σ_C [C]	G[%]	σ_C [G]	T[%]	σ_C [T]
	2	25.71	5.54	24.38	5.28	24.65	4.95	24.90	5.20	2	42.55	14.55	22.81	13.48	25.01	14.13	40.47	13.18
	4	25.36	5.06	24.80	5.17	24.73	4.95	24.87	5.67	4	36.73	20.02	21.26	13.98	21.86	14.10	31.97	18.47
$k=1$	6	26.11	5.08	24.49	4.16	24.23	4.08	24.95	4.30	6	33.41	18.97	20.95	13.48	21.45	13.36	28.45	17.62
	8	25.49	4.55	24.52	4.19	24.35	4.18	25.43	4.50	8	32.92	16.95	20.01	10.97	21.56	11.11	27.60	16.08
	10	25.35	4.09	24.91	4.04	24.65	4.14	24.90	3.96	10	32.53	15.32	19.32	10.33	21.74	10.80	27.25	15.02
	2	27.62	5.13	21.78	5.09	22.77	5.48	27.83	5.36	2	31.93	11.44	22.82	10.61	21.59	10.29	31.88	12.57
	4	27.63	4.99	22.07	5.00	22.28	4.50	28.02	4.99	4	29.46	11.69	21.76	9.87	21.95	9.81	29.95	12.44
$k=2$	6	27.95	5.91	22.24	5.39	21.96	5.11	27.84	4.96	6	28.09	11.62	21.76	9.12	21.62	8.95	29.66	12.16
	8	27.62	4.73	22.16	4.45	22.20	4.36	28.02	4.94	8	28.07	11.33	21.32	9.83	21.95	10.62	29.45	12.58
	10	27.57	4.64	22.17	4.19	22.27	4.01	27.99	4.34	10	27.79	11.30	21.26	9.22	21.42	9.55	29.65	12.03
	2	28.52	6.19	22.41	4.87	21.39	4.51	27.67	5.12	2	31.20	7.92	19.97	6.75	19.63	5.92	29.20	7.57
	4	28.19	5.44	22.51	4.84	21.68	4.80	27.62	4.97	4	31.45	8.15	20.03	5.94	19.84	5.90	29.06	7.43
$k=3$	6	27.83	5.20	22.68	4.89	21.71	4.45	27.78	4.89	6	30.93	6.62	19.89	5.88	20.03	5.79	29.30	7.17
	8	27.86	5.13	22.46	4.63	21.74	4.43	27.94	4.70	8	31.34	6.30	19.54	5.79	19.90	5.49	29.36	6.48
	10	27.61	4.83	22.71	4.22	21.89	4.03	27.80	4.42	10	31.25	5.79	19.48	5.72	19.64	5.51	29.63	6.21

	CHROMOSOME 19									CHROMOSOME 20								
	n	A [%]	σ_C [A]	C[%]	σ_C [C]	G[%]	σ_C [G]	T[%]	σ_C [T]	n	A [%]	σ_C [A]	C[%]	σ_C [C]	G[%]	σ_C [G]	T[%]	σ_C [T]
	2	38.68	16.90	25.41	13.50	23.06	11.51	38.36	14.56	2	35.49	13.10	24.93	12.17	21.37	12.11	41.67	15.11
	4	32.65	17.90	21.61	14.88	23.15	13.99	30.64	17.10	4	29.01	17.31	24.09	13.86	19.02	14.33	36.45	18.70
$k=1$	6	30.43	18.05	20.61	15.50	24.70	15.61	26.28	17.62	6	27.49	16.91	23.23	13.43	19.70	14.02	32.51	18.17
	8	29.95	18.15	20.73	15.20	24.61	15.06	25.99	17.05	8	25.65	16.25	24.03	13.52	19.20	14.16	32.86	17.67
	10	29.30	16.57	21.25	14.76	24.64	15.24	25.51	16.54	10	26.47	15.40	22.98	12.55	20.00	13.28	31.47	16.89
	2	38.61	14.65	20.05	11.18	25.03	13.78	36.39	14.68	2	37.88	14.25	23.84	13.07	22.79	11.18	38.59	14.92
	4	34.50	16.28	19.97	12.87	25.59	14.52	28.19	17.72	4	31.75	18.28	22.38	14.30	20.80	12.67	33.24	18.42
$k=2$	6	30.68	16.89	21.23	13.41	25.19	15.09	26.28	17.61	6	29.21	17.20	21.65	14.26	21.59	14.24	31.39	18.24
	8	29.23	15.55	21.85	12.49	24.95	13.11	25.28	15.67	8	27.95	13.06	22.28	11.21	23.55	11.85	26.91	13.73
	10	27.52	15.23	23.16	12.88	25.04	12.76	24.72	14.68	10	28.68	15.74	21.82	13.93	20.67	13.14	29.55	15.70
	2	35.86	13.33	25.37	13.36	23.27	13.08	38.80	15.55	2	33.43	12.67	22.50	10.70	23.62	9.91	32.60	15.17
	4	29.55	16.31	24.42	14.64	22.30	14.52	31.97	17.94	4	31.75	18.28	22.38	14.30	20.80	12.67	33.24	18.42
$k=3$	6	26.12	17.33	25.32	15.54	22.15	15.52	29.65	17.41	6	28.45	14.39	21.95	11.96	22.89	12.12	28.57	14.46
	8	25.09	16.78	25.20	15.10	22.89	14.72	28.67	17.03	8	27.95	13.06	22.28	11.21	23.55	11.85	26.91	13.73
	10	24.33	14.52	25.66	14.61	24.01	14.61	26.30	14.77	10	27.78	12.94	22.06	11.55	23.88	11.58	26.63	13.24

Table 5: Same as Table 1 but for the chromosomes 17, 18, 19, 20.

	CHROMOSOME 21									CHROMOSOME 22								
	n	A [%]	σ_C [A]	C[%]	σ_C [C]	G[%]	σ_C [G]	T[%]	σ_C [T]	n	A [%]	σ_C [A]	C[%]	σ_C [C]	G[%]	σ_C [G]	T[%]	σ_C [T]
$k = 1$	2	32.52	9.24	19.66	7.38	21.05	7.01	28.31	9.95	2	28.21	8.29	23.37	5.53	24.47	5.44	25.65	9.69
	4	32.27	10.84	19.25	7.69	21.18	8.50	27.29	10.81	4	28.30	8.40	22.93	5.81	24.63	5.53	24.92	8.48
	6	32.20	10.90	18.38	8.19	20.98	8.07	28.45	10.73	6	27.59	8.41	23.98	6.54	24.10	5.87	24.81	7.87
	8	32.41	10.32	17.72	7.97	21.53	8.50	28.44	10.97	8	26.79	7.54	24.46	7.00	23.85	6.70	25.19	7.22
	10	33.28	9.08	16.28	7.82	23.14	9.14	27.29	9.70	10	26.91	7.33	24.28	7.20	23.32	7.10	25.91	7.13
$k = 2$	2	31.33	12.48	20.18	8.33	21.33	8.27	27.94	12.05	2	28.72	7.15	23.14	6.23	22.19	6.52	26.81	6.35
	4	30.79	12.03	20.27	8.92	21.61	8.30	27.61	12.33	4	28.05	6.28	22.47	5.96	22.82	6.22	26.80	7.09
	6	31.16	11.19	19.70	7.91	20.83	7.86	28.42	11.55	6	28.21	6.46	22.49	7.13	23.53	7.29	26.03	7.54
	8	31.73	10.24	19.58	7.46	20.48	7.80	28.21	11.21	8	28.16	6.65	22.20	7.20	23.21	7.10	26.44	7.23
	10	31.96	9.91	19.43	7.70	20.50	7.75	28.27	10.63	10	27.97	5.58	22.28	5.32	23.39	6.14	26.35	6.65
$k = 3$	2	28.33	10.45	22.50	9.69	24.70	8.74	25.18	9.31	2	25.65	6.75	25.32	7.10	23.36	6.74	25.83	8.30
	4	28.38	10.78	22.33	9.23	24.33	9.51	25.69	9.84	4	25.56	6.21	25.26	6.48	23.93	6.33	25.74	7.70
	6	27.95	9.06	21.98	8.10	24.01	8.76	26.06	9.32	6	25.55	6.23	25.26	6.02	24.39	6.13	24.94	6.98
	8	28.36	8.95	21.66	7.86	24.10	8.33	25.87	8.83	8	24.90	5.56	25.50	5.78	24.92	6.67	24.67	6.40
	10	27.94	8.86	21.64	7.85	24.30	9.44	26.31	8.78	10	24.71	5.89	25.73	6.63	25.08	6.38	24.49	6.03

	CHROMOSOME X									CHROMOSOME Y								
	n	A [%]	σ_C [A]	C[%]	σ_C [C]	G[%]	σ_C [G]	T[%]	σ_C [T]	n	A [%]	σ_C [A]	C[%]	σ_C [C]	G[%]	σ_C [G]	T[%]	σ_C [T]
$k = 1$	2	38.67	14.73	25.21	11.59	26.64	13.51	37.66	15.49	2	27.56	5.91	21.89	5.63	23.92	6.35	27.13	7.52
	4	32.88	18.06	20.86	14.09	25.39	14.87	28.01	19.06	4	27.45	6.19	22.05	6.66	24.21	7.08	26.49	7.47
	6	31.16	17.92	20.53	14.69	24.35	15.84	27.09	18.84	6	27.99	6.64	21.59	6.55	24.40	7.23	26.02	7.50
	8	30.65	18.29	20.27	15.59	24.21	15.68	26.05	18.37	8	28.12	7.02	21.55	7.17	24.14	7.85	26.19	8.48
	10	30.12	17.79	20.48	14.47	22.97	15.27	27.38	18.34	10	27.89	6.99	21.88	6.60	23.30	7.52	26.90	8.10
$k = 2$	2	41.63	15.41	20.02	10.78	25.17	14.33	38.13	13.19	2	31.50	5.49	19.11	3.89	19.90	5.15	29.49	5.97
	4	36.55	19.80	19.34	14.29	23.76	14.87	30.55	18.56	4	31.25	5.32	19.39	4.36	19.72	4.72	29.64	5.89
	6	34.19	17.37	18.30	13.05	24.36	14.39	26.35	17.62	6	31.00	5.51	19.38	4.23	20.45	4.93	29.17	5.71
	8	32.40	17.61	18.84	12.59	23.18	13.59	26.49	17.30	8	30.82	5.48	19.57	4.31	20.34	4.65	29.28	5.38
	10	31.63	15.34	18.49	11.41	23.45	13.08	26.43	15.56	10	30.83	5.16	19.28	4.26	20.68	4.24	29.20	4.85
$k = 3$	2	40.67	14.60	23.74	14.84	22.29	13.32	39.75	13.89	2	31.33	5.54	19.29	4.94	20.09	4.91	29.29	6.08
	4	33.73	17.43	21.57	12.97	21.01	13.04	32.29	15.92	4	31.61	6.17	18.90	4.98	20.53	5.72	28.96	6.95
	6	29.39	17.92	22.04	13.80	19.14	12.31	31.79	16.95	6	30.91	5.64	19.35	4.42	20.25	4.41	29.49	5.88
	8	29.00	17.57	21.27	12.02	19.26	11.20	32.49	15.59	8	30.76	5.34	19.29	3.98	20.34	4.47	29.60	6.02
	10	28.61	16.07	20.97	11.35	19.76	10.58	31.25	15.08	10	30.61	5.37	19.19	4.15	20.46	4.59	29.73	5.77

Table 6: Same as Table 1 but for the chromosomes 21, 22, 23, 24.
